# A novel hotspot specific isothermal amplification method for detection of the common *PIK3CA* p.H1047R breast cancer mutation

**DOI:** 10.1038/s41598-020-60852-3

**Published:** 2020-03-12

**Authors:** Melpomeni Kalofonou, Kenny Malpartida-Cardenas, George Alexandrou, Jesus Rodriguez-Manzano, Ling-Shan Yu, Nicholas Miscourides, Rebecca Allsopp, Kelly L. T. Gleason, Katie Goddard, Daniel Fernandez-Garcia, Karen Page, Pantelis Georgiou, Simak Ali, R. Charles Coombes, Jacqueline Shaw, Christofer Toumazou

**Affiliations:** 1 Centre for Bio-Inspired Technology, Department of Electrical and Electronic Engineering, Imperial College London, London, SW7 2AZ England; 2 Leicester Cancer Research Centre, Department of Genetics and Genome Biology, University of Leicester, Leicester, LE2 7LX England; 3 Division of Cancer, Department of Surgery and Cancer, Imperial College London, London, SW7 2AZ England

**Keywords:** Breast cancer, DNA, Molecular biology

## Abstract

Breast cancer (BC) is a common cancer in women worldwide. Despite advances in treatment, up to 30% of women eventually relapse and die of metastatic breast cancer. Liquid biopsy analysis of circulating cell-free DNA fragments in the patients’ blood can monitor clonality and evolving mutations as a surrogate for tumour biopsy. Next generation sequencing platforms and digital droplet PCR can be used to profile circulating tumour DNA from liquid biopsies; however, they are expensive and time consuming for clinical use. Here, we report a novel strategy with proof-of-concept data that supports the usage of loop-mediated isothermal amplification (LAMP) to detect *PIK3CA* c.3140 A > G (H1047R), a prevalent BC missense mutation that is attributed to BC tumour growth. Allele-specific primers were designed and optimized to detect the p.H1047R variant following the USS-sbLAMP method. The assay was developed with synthetic DNA templates and validated with DNA from two breast cancer cell-lines and two patient tumour tissue samples through a qPCR instrument and finally piloted on an ISFET enabled microchip. This work sets a foundation for BC mutational profiling on a Lab-on-Chip device, to help the early detection of patient relapse and to monitor efficacy of systemic therapies for personalised cancer patient management.

## Introduction

Breast cancer (BC) has a lifetime incidence risk of 12%, with an overall survival of more than 70% of reported cases when early detection is possible^1,2^. Although surgery is capable of removing the primary tumour, minimal residual disease (MRD)/micrometastases may persist resulting in eventual resistance to therapy and recurrence^3^. MRD can often persist even after adjuvant therapy and can grow and spread overtime, remaining undetectable through mammograms, MRI scans and current tumour marker blood tests, such as CA-15-3 and CA 27.29 antigen assays^4^. These current tumour marker blood tests work as a cost-effective method to measure disease progression but do not provide information about mutational changes and heterogeneity of the primary tumour or MRD. With the development of new, non-cross-resistant treatments for breast cancer, early detection of MRD/micrometastases and mutational profiling of circulating tumour DNA (ctDNA) provides an attractive, cost-effective alternative approach to the detection of early signs of relapse and treatment switching.

Previous research has proven that circulating free DNA (cfDNA) contains DNA fragments from cancer cells (ctDNA) that are released in blood, showing somatic mutations that reflect the original cancer and evolved clonal subtypes^5^. In particular, recent efforts have established that hotspot mutations in *PIK3CA*, a gene mutated in 20–40% of metastatic BC, are also commonly observed in screen-detected stage-1 BCs^6^. The PI3K protein is involved in the AKT/mTOR pathway and, when deregulated, leads to tumour-cell growth^7^. Investigation has indicated that the PIK*3CA* p.H1047R missense mutation causes a constitutively active form of the PI3K protein and is associated with poor prognosis and BC disease progression^8^. Detecting BC mutations from ctDNA has been successful with next-generation sequencing workflows like BEAMing^9–11^ or digital droplet PCR (ddPCR) but cannot be easily translated into the clinic due to multifactorial reasons such as requirement of clear prognostic value, expensive costs and specialised labour per sample^5,12,13^.

**Figure 1:**
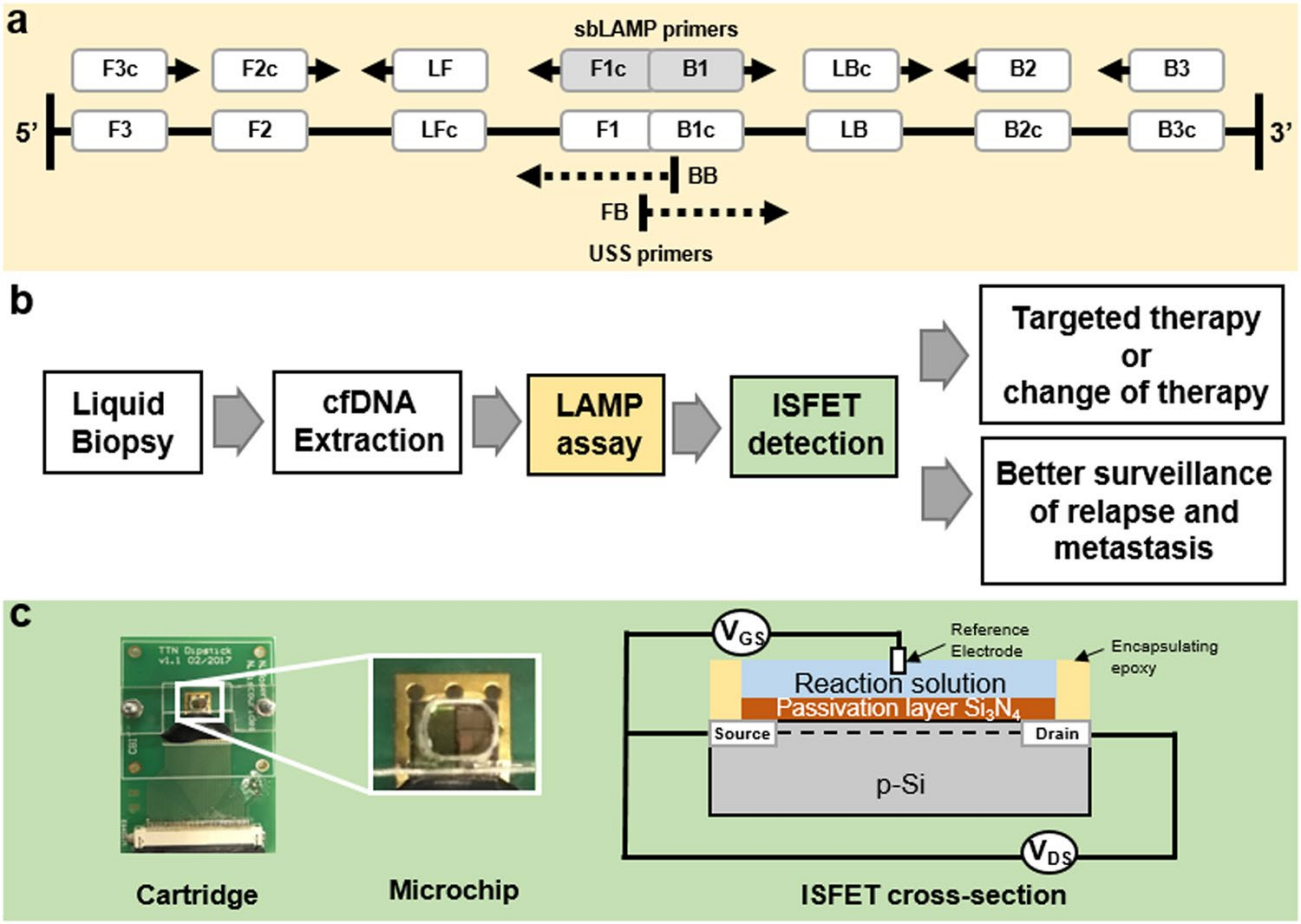
Schematic of the SNV-based LAMP method, proposed workflow and cartridge of CMOS microchip. **(a)** Representation of the SNV-based LAMP method including USS primers that prevent unspecific amplification^15^. **(b)** Proposed workflow showing how the protocol described here can be implemented with liquid biopsies extraction to patient’s treatments and relapses management. **(c)** Lab-on-Chip platform showing the PCB cartridge, microchip, microfluidic chamber and cross-section of an ISFET sensor^16^.

To circumvent this requirement of expensive thermal-cycling machinery of PCR technology and sequencing workflows, isothermal amplification was investigated as an alternative method of detection. Loop-mediated isothermal amplification (LAMP) developed by *Notomi et al*.^14^ uses 6 template specific primers to amplify DNA with high specificity and sensitivity at a constant temperature. LAMP has often been used in the study of pathogens and infectious diseases but has recently started to be used for mutational detection. A variation of the LAMP protocol using USS-sbLAMP primers^15^ can help discriminate single base mutational detection allowing cancer variants to be investigated by LAMP, as shown in Fig. 1a. These properties of LAMP coupled with its higher amplification yield allows it to be utilised in tandem with standard microchip technology used for chemical sensing such as the Ion-Sensitive Field-Effect Transistors (ISFETs)^16^. ISFETs fabricated in unmodified CMOS technology have been already described to detect DNA targets via the pH change induced during positive amplification results^17–23^. ISFET-based DNA detection in combination with LAMP’s ability to run isothermally allows the technology to be applied at the point-of-care as a Lab-on-Chip (LoC) device.

In this study, novel primer sets for the detection of *PIK3CA* p.H1047R variant in isothermal conditions have been developed, enabling fast, sensitive and specific detection of wild type (WT) and mutant (MT) p.H1047R alleles, while differentiating the detectable signals in an environment of interfering DNA templates. Time-to-results below 25 minutes with a limit of detection of 10^3^ copies per reaction show the potential of adapting this assay to the clinic for rapid and sensitive profiling of common BC mutations. Besides lab-based validation, we demonstrate on-chip specific detection of both WT and MT alleles using an ISFET-based Lab-on-Chip platform. Using this fully-electronic chemical sensing technology in combination with the molecular methods developed here, we are in a position to create a lab-free, cost-effective sample-to-result system for routine monitoring of a panel of breast cancer mutations through liquid biopsy profiling of ctDNA, to be used as a diagnostic companion to stratify patient treatment. Future adoption would lead to significant advantages, such as offering continuous monitoring of tumour progression while assisting imaging intervention and tumour localisation, aiming to adaptively individualise breast cancer treatment for patient benefit. The technology would also be primed to monitor MRD post-surgery and help proactively search for therapy resistance mutations during a patients’ treatment regime. The proposed workflow of the system and the Lab-on-Chip platform used to demonstrate feasibility of the SNV-based LAMP method are demonstrated in Fig. 1(b,c).

## Results

### SNV-specific LAMP for detection of p.H1047R

Specific amplification of the *PIK3CA* p.H1047R gene mutation in isothermal conditions was achieved following the USS-sbLAMP method described by *Malpartida-Cardenas et al*.^15^. This method consisted of (i) Single Nucleotide Variant (SNV) LAMP (sbLAMP) primers in charge of allele-specific amplification, and (ii) unmodified self-stabilising (USS) competitive primers responsible for preventing unspecific amplification. The sbLAMP primers included the LAMP priming regions F3, B3, LF, LB and the allele-specific primers sbFIP and sbBIP. These last two primers contained the regions F1c-F2 and B1c-B2, respectively. Considering the GC% content of the target sequence, different lengths of F1c and B1c priming regions were designed for optimal performance at 63 °C and screened experimentally; due to the higher GC% content at the region where B1c is located, the final design consisted of B1c being shorter than F1c to enhance allele-specificity without hampering the analytical sensitivity.

Specifically, the sbLAMP primer set targeting the mutant (MT) allele, named as sbLAMP_MT,_ included sbFIP_17__MT and sbBIP_14__MT primers. On the other hand, the sbLAMP primer set targeting the wild type (WT) allele, named as sbLAMP_WT,_ consisted of sbFIP_19__WT and sbBIP_14__WT primers. USS primers were designed manually based on the aforementioned sbFIP and sbBIP primers. USS primers were incorporated in the reaction as follows: MT reaction included sbLAMP_MT_ with USS_WT_, and WT reaction included sbLAMP_MT_ with USS_WT_. Different lengths and concentrations were tested following the guidelines described^15^. The final primer sets consisted of USS_MT_ FB2_21__MT/BB2_24__MT and USS_WT_ FB2_19__WT/BB1_23__WT, both at a concentration of 3 µM per reaction. Primer sequences are shown in Table 1.

**Table 1:** USS-sbLAMP primer sequences for allele-specific amplification of SNV p.H1047R.

Primer ID	Sequence (5′→ 3′)
H1047R_F3	AGA ACT ACA ATC TTT TGA TGA CA
H1047R_B3	TGG AAT CCA GAG TGA GCT
H1047R_LF	AAT ACT CCA AAG CCT CTT GCT C
H1047R_LB	TGG ATC TTC CAC ACA ATT AAA CAG C
H1047R_sbFIP_19__WT	TGT GCA TCA TTC ATT TGT TGC ATA CAT TCG AAA GAC CCT
H1047R_sbBIP_14__WT	ATC ATG GTG GCT GGC TCA GTT ATC TTT TCA GTT CAA TG
H1047R_sbFIP_17__MT	CGT GCA TCA TTC ATT TGG CAT ACA TTC GAA AGA CCC T
H1047R_sbBIP_14__MT	GTC ATG GTG GCT GGC TCA GTT ATC TTT TCA GTT CAA TG
H1047R_FB2_21__MT	ACG TCA TGG TGG CTG GAC AAC AA
H1047R_BB2_24__MT	GAC GTG CAT CAT TCA TTT GTT TCA TG
H1047R_FB2_19__WT	ACA TCA TGG TGG CTG GAC AAC
H1047R_BB1_23__WT	ATG TGC ATC ATT CAT TTG TTT CAT

### Analytical sensitivity and specificity of SNV-specific LAMP

**Figure 2:**
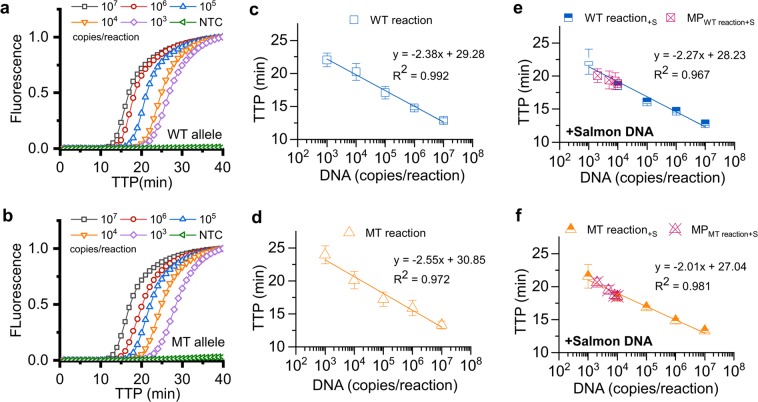
Amplification and standard curves of USS-sbLAMP reactions for detection of SNV p.H1047R utilising a synthetic plasmid DNA template containing a 531 bp fragment of the gene region. **(a)** Amplification curves of the WT template at different DNA concentrations ranging from 10^7^ to 10^3^ copies/reaction (31.356 pg/reaction to 0.003 pg/reaction respectively) using the WT reaction. **(b)** Amplification curves of the MT template at different DNA concentrations ranging from 10^7^ to 10^3^ copies/reaction (31.356 pg/reaction to 0.003 pg/reaction respectively) using the MT reaction. **(c)** Standard curve of the DNA template harbouring the WT allele, showing linear fitting equation and coefficient of determination. **(d)** Standard curve of the DNA template harbouring the MT allele, showing linear fitting equation and coefficient of determination. **(e)** Standard curve of the DNA template harbouring the WT allele with the addition of background Salmon testes sonicated DNA at 13 ng/reaction (+S). Results from the specific amplification of mixed populations are depicted in dark pink and labelled as MP_WT reaction+S_. **(f)** Standard curve of the DNA template harbouring the MT allele with the addition of background Salmon testes sonicated DNA at 13 ng/reaction (+S). Results from the specific amplification of mixed populations are depicted in dark pink and labelled as MP_MT reaction+S_. Error bars are displayed at one standard deviation. Reactions were considered as negative at time-to-positive (TTP) above 25 min. No template control (NTC) was included. Average of two experiments performed in triplicates.

Analytical sensitivity of the designed LAMP primer sets was evaluated with ten-fold serial dilutions of synthetic plasmid DNA at 1 × 10^7^, 1 × 10^6^, 1 × 10^5^, 1 × 10^4^ and 1 × 10^3^ copies/reaction. Two reactions, WT and MT reactions, were performed independently. WT and MT templates were tested in both reactions to assess the analytical sensitivity and specificity of the designed USS-sbLAMP primer sets. Both templates were uniquely amplified within their corresponding specific reactions at concentrations below 1 × 10^6^ copies/reaction with a limit of detection of 1 × 10^3^ copies/reaction within 25 min. In Fig. 2a, standard curves are presented showing coefficient of determinations (R^2^) of 0.992 and 0.972 for the specific amplification of the WT and MT template, respectively. These results showed the capability of the assay for sample quantification. Both WT and MT reactions presented a linear working range from 1 × 10^7^ to 1 × 10^3^ copies/reaction. A complex and more realistic genomic environment was created by incorporating Salmon testes sonicated DNA in the reaction mixture as background DNA. Results presented in Fig. 2b show standard curves of specific WT and MT reactions. Remarkably, the limit of detection (1 × 10^3^ copies/reaction) and the linear working range (1 × 10^7^ to 1 × 10^3^ copies/reaction) were preserved in this scenario without any significant variation in the TTP values (correlation of 0.98 and 0.99 for each specific reaction, according to statistical linear regression), as shown in Table 2.

**Table 2:** TTP values of standard curves of USS-sbLAMP reactions for detection of SNV p.H1047R.

DNA $(\frac{{\boldsymbol{copies}}}{{\boldsymbol{reaction}}})$	− Salmon DNA^a^				+ Salmon DNA^b^			
	WT template (TTP ± SD)		MT template (TTP ± SD)		WT template (TTP ± SD)		MT template (TTP ± SD)	
	WT reaction	MT reaction	WT reaction	MT reaction	WT reaction	MT reaction	WT reaction	MT reaction
1 × 10^7^	12.77 ± 0.24	17.62 ± 0.32	18.92 ± 0.61	13.29 ± 0.25	12.87 ± 0.40	19.19 ± 0.63	18.21 ± 0.26	13.33 ± 0.60
1 × 10^6^	14.67 ± 0.22	20.98 ± 1.36	22.21 ± 2.18	14.81 ± 0.44	14.75 ± 0.28	22.85 ± 1.89	21.27 ± 0.61	15.90 ± 1.17
1 × 10^5^	16.1 ± 0.28	NEG	NEG	16.81 ± 0.27	17.07 ± 0.97	NEG	NEG	17.23 ± 1.07
1 × 10^4^	18.57 ± 0.64	NEG	NEG	18.43 ± 0.66	20.22 ± 1.30	NEG	NEG	20.13 ± 1.30
1 × 10^3^	22.19 ± 1.9	NEG	NEG	21.54 ± 1.79	22.03 ± 1.04	NEG	NEG	23.96 ± 1.38

^a^TTP values of WT and MT reactions with WT and MT template.

^b^TTP values of WT and MT reactions with WT and MT template, including Salmon DNA as background at 13 ng/reaction.

Reactions were considered as negative at TTP above 25 min.

### Accuracy of detection in a mixture of allele ratios

**Figure 3:**
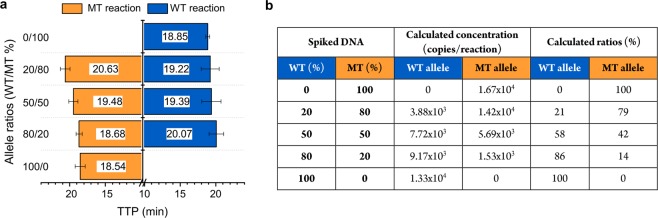
Study of SNV p.H1047R mix populations including wild-type and mutant alleles at different ratios. **(a)** TTP values of the spiked mixed populations at different ratios when tested with the WT and MT reactions, independently. **(b)** Calculated concentrations using the equations from the standard curves previously derived in Fig. 2, and calculated ratios using equation $(\frac{{{\rm{x}}}_{1}}{{{\rm{x}}}_{1}+{{\rm{y}}}_{1}})\times 100$ where *x*_1_ and *y*_1_ represent the calculated concentration of either the WT or MT allele. Mixtures were prepared to a final concentration of 10^4^ copies/reaction (100%). Spiked mixed populations at different ratios expressed in percentages (100/0, 80/20, 50/50, 20/80, and 0/100).

Allele discrimination within allelic ratios (100/0, 80/20, 50/50, 20/80, and 0/100, in percentages) in spiked mixed populations was studied. This scenario included Salmon testes sonicated background DNA with the results shown in Fig. 3. Both alleles were discriminated by combining the results of the WT and MT reactions, denoting the high specificity of the assay to distinguish each allele within mixed populations. Reactions targeting the allele that was not present in the mixed population, such as the ratios 100/0 and 0/100, did not show any amplification. The TTP values obtained clearly fitted in the corresponding standard curves previously built for each allele (Fig. 2b), and therefore, the capability of DNA quantification within a mixed population was demonstrated. Calculated concentrations in copies/reaction and the calculated ratios are presented in Fig. 3b. Overall, these results show the feasibility of the developed assay to estimate copy number and ratios with high accuracy.

### Validation with positive and negative DNA samples derived from patient tissue and cell line controls

**Figure 4:**
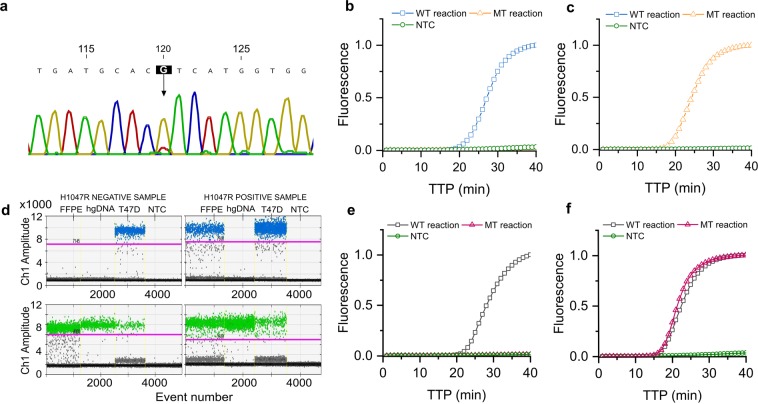
Amplification curves of USS-sbLAMP reactions for detection of SNV p.H1047R in T47D cell lines and Formalin-Fixed Paraffin-Embedded (FFPE) tissue. **(a)** Validation of p.H1047R mutation in T47D cell lines using Sanger Sequencing. High predominance of nucleotide “G” in the heterozygous cell line. **(b)** MCF7 cell line harbouring the WT p.H1047R allele tested with WT and MT reactions, independently. **(c)** T47D heterozygous cell line tested with WT and MT reactions, independently. **(d)** ddPCR validation of p.H1047R mutation in FFPE tissue derived DNA samples. DNA samples included 10 ng of FFPE DNA (FFPE), 10 ng of hgDNA as negative control (hgDNA), 10 ng of T47D cell line as heterozygous control (T47D) and no template control (NTC). Representative one-dimensional (1D) amplitude plots are shown with the associated manual threshold gating (pink line) for FAM (mutant, blue) amplitudes in upper panels and VIC (wild type, green) amplitudes in lower panels. Black dots correspond to droplets without any dye fluorescence. Left hand panel shows FFPE tissue DNA negative for c.3140 A > G mutation and right-hand panel shows FFPE tissue DNA heterozygous for the c.3140 A > G mutation. **(e)** FFPE tissue DNA harbouring the WT p.H1047R allele was tested with WT and MT reactions, independently. **(f)** FFPE tissue heterozygous DNA harbouring both WT and MT alleles was tested with WT and MT reactions, independently.

DNA from MCF7, T47D cell lines (Fig. 4a–c) and from Formalin-Fixed Paraffin-Embedded (FFPE) patient tumour tissue cores (Fig. 4d–f) were assessed as positive and negative controls for the p.H1047R mutation using the developed USS-sbLAMP assay. The T47D cell line DNA was analysed by Sanger sequencing (Fig. 4a), showing the high predominance of the nucleotide “G” over nucleotide “A”. Amplification curves are shown in Fig. 4b,c, corroborating the specificity and accuracy of the developed assay to amplify the corresponding target; the T47D cell line was uniquely amplified with the MT reaction indicating that the presence of the WT allele was out of the limit of detection to be amplified by the WT reaction. The MCF7 cell line was uniquely amplified with the WT reaction as expected. The obtained TTP values corresponded to approximately 1.90 × 10^3^ copies/reaction (MCF7 cell line) and 1.53 × 10^4^ copies/reaction (T47D cell line) according to the standard curves previously built for the WT and MT allele respectively.

DNA extracted from two patient FFPE tissue cores were used as controls for wild type (healthy tissue core) and heterozygous mutant (tumour tissue core) status respectively. Presence or absence of the p.H1047R mutation was confirmed by ddPCR (Fig. 4d). FFPE tissue DNA negative for the p.H1047R mutation (left hand panels in Fig. 4d) showed an allele fraction of 0% c.3140 A > G MT allele. The heterozygous sample (right hand panels in Fig. 4d) showed an allele fraction of 38.2% c.3140 A > G MT allele and 61.8% c.3140 A WT allele. The WT sample (FFPE tissue DNA negative for p.H1047R) uniquely amplified with the WT reaction (Fig. 4e), whilst the heterozygous sample (FFPE tissue DNA positive for p.H1047R) amplified with both reactions obtaining similar TTP values (Fig. 4f). The TTP values obtained with the FFPE tissue DNA WT sample corresponded to approximately 1.70 × 10^2^ copies/reaction. In the case of the heterozygous sample, TTP values obtained with the WT reaction corresponded to 6.06 × 10^4^ copies/reaction of the WT allele and TTP values obtained with the MT reaction corresponded to 1.04 × 10^5^ copies/reaction of the MT allele. These results reinforce the capability of the developed assay to discriminate the SNV p.H1047R at isothermal conditions with high accuracy in FFPE tumour tissues, even in the presence of mixed populations.

### Adaptation of SNV-specific LAMP on a Lab-on-Chip platform

**Figure 5:**
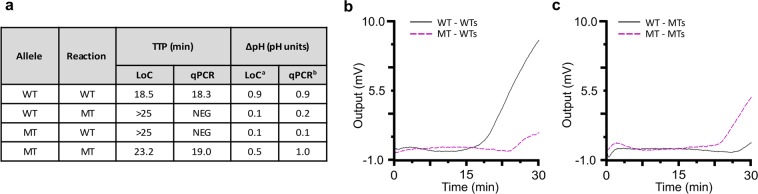
Lab-on-Chip results for the detection of SNV p.H1047R. **(a)** TTP values and ΔpH measurements of reactions performed on the Lab-on-Chip platform and conventional qPCR instrument. **(b)** Amplification curves of the WT template with WT and MT reactions carried-out independently on the Lab-on-Chip platform. **(c)** Amplification curves of the MT template with WT and MT reactions carried-out independently on the Lab-on-Chip platform. ^a^Equivalent pH based on microchip sensitivity (−9.23 mV/pH). ^b^pH measurements with a pH-meter (Sentron).

The developed WT and MT reactions were tested using an ISFET-based Lab-on-Chip platform^16,18^ with synthetic DNA harbouring either the WT or MT allele to prove the feasible translation of the lab-based assay at a point-of-care setting. The reaction conditions were modified such that pH changes could occur during DNA amplification. This consisted of changing the buffering conditions in LAMP to those described in pH-LAMP^20^. Each synthetic sample was tested with each of the WT and MT reactions independently for 25 min at a constant temperature of 63 °C (provided by a thermal controller). As shown in Fig. 5, each reaction amplified the sample harbouring the targeted allele, as previously shown with the qPCR instrument. The amplification curves obtained on-chip are shown in Fig. 5b, and the TTP values were in agreement with the values obtained with the qPCR instrument. Besides the correlation of the TTP values, pH measurements of the reactions carried out on-chip and with the bulky instrument were also similar, as described in Fig. 5a. Consequently, we demonstrate a proof-of-concept that the developed assays can be safely transferred to the LoC platform for lab-free SNV detection of the mutation p.H1047R related to BC (Fig. 5).

## Discussion

In this study, we report two novel primer sets using an allele-specific LAMP assay to detect the missense p.H1047R, *PIK3CA* somatic mutation, which is a common driver mutation in breast cancer. *PIK3CA* is also one of the most frequently mutated genes in human breast cancer and in numerous other malignancies^24^ due to the cellular importance of the PI3K signalling cascade. The potential clinical utility of being able to easily and rapidly determine *PIK3CA* mutations is yet to be fully explored. The SOLAR-1 trial recently presented that using combination therapy with Alpelisib for patients with an oncogenic *PIK3CA* mutation showed nearly double progression free survival^25^. Therefore, this assay could potentially be used to analyse diagnostic core biopsy tissue to identify a potential option for targeted monotherapy or combination therapies, with the recently approved small molecule inhibitor Alpelisib. Moreover, *PIK3CA* mutation is also associated with decreased effectivity of other common drugs such as Trastuzumab^26^. Therefore, detection of *PIK3CA* mutation could enable a precision medicine approach to determine the most appropriate therapy. These are two examples where a single mutation being identified can aid clinicians in their decision-making. Multiplexing this system where the LoC platform can detect panels of mutations would thus be a highly beneficial aid to clinicians in determining course of action as well as potentially monitoring MRD and relapse in BC patients.

The variant specific LAMP primer sets were designed to discriminate each allele by delaying or preventing unspecific amplification. At DNA concentrations below 1 × 10^6^ copies/reaction of synthetic template, each primer set uniquely amplified the targeted allele. Analytical specificity and sensitivity were preserved in the presence of background Salmon DNA with a limit of detection of 1 × 10^3^ copies/reaction, which equates to 0.003 pg/reaction for the synthetic template. Furthermore, allele discrimination within allelic ratios in spiked mixed populations was demonstrated enabling quantification with values within 8% from the reference ones. As a proof-of-concept, DNA samples from two breast cancer cell lines and FFPE tumour tissues were tested with the developed assay. The results obtained, reinforced the robustness, sensitivity and specificity of the developed assay for the discrimination of p.H1047R in cell lines and patient tissue samples. In addition, we show allelic discrimination using a pH sensitive variation of the proposed assay with a CMOS microchip (Fig. 5)^16^. The microchip consisted of an array of ISFET sensors which detected both alleles with a positive change in pH of around 1–1.48 pH units. Overall, we show novel sbLAMP primer sets with the potential to discriminate BC tissue samples carrying the mutation p.H1047R as well as detection with CMOS-based ISFET sensors. This may provide the basis to develop an alternative to blood-based mutation testing by NGS and ddPCR technology in a quicker and more affordable way for use with plasma-derived cfDNA.

Other studies have reported the capability of detecting the mutation p.H1047R. *Alvarez-Garcia et al*.^27^ genotyped *PIK3CA* status with an analytical sensitivity of 56 pg (9 genome equivalents) using qPCR technology and chemically modified primers. In a similar way, *Wang et al*.^28^ used non-extendable primer blockers for allele-specific PCR detection of three mutations in cancer *K-RAS, B-RAF*, and *EGFR* with a limit of detection of a single copy. A different approach reported by *Gyanchandani et al*.^29^, has been used to demonstrate amplification of cfDNA from liquid biopsies linked to metastatic BC without compromising allelic balance. This step enriched the cfDNA sample, allowing ddPCR and sequencing to become more feasible due to the higher amount of DNA template required. Numerous NGS technologies such as BEAMing and Safe-SeqS have been used to detect known breast cancer mutations using ctDNA with sensitivity higher than 99%^30^. Our own studies have shown for primary patients that recurred, patient specific ctDNA profiling detected molecular relapse up to 2 years ahead of clinical relapse with 89% sensitivity and 100% specificity^31^. However, despite its high sensitivity and specificity, NGS platforms are still costly and require specialist time for data analysis and experimentation. As such, NGS can be useful to discover tumour mutations that can aid in treatment decisions and cancer classifications but this technique is not currently used routinely to monitor cancer response to treatment. On the other hand, our isothermal assay and Lab-on-Chip platform may allow for rapid, affordable and portable monitoring of individual breast cancer mutations without the need of thermal cycling, chemically modified primers or bulky and expensive equipment. Going forward, the next step will be to develop a multiplex assay to survey several common breast cancer mutations. The cost-effectiveness of such a test will allow breast cancer to be monitored routinely during patient’s therapeutic journey.

LAMP has been previously used to detect mutations in tumour tissue such as for *KRAS* mutations in colorectal colon cancer^32^ using LAMP in tandem with ligation substrates. Two studies specific to the detection of *PIK3CA* mutations published recently involved a colorimetric assay using strand displacement amplification^33^, and recombinase polymerase amplification (RPA), which is another isothermal amplification method^34^. These other studies highlight the demand for a cost-effective and rapid method for mutational tracking of DNA markers in cancer. In this paper, we demonstrate the genotyping of breast cancer variants with isothermal methods and CMOS technology, specifically detecting the *PIK3CA* p.H1047R variant. The combination of these principles indicates the feasibility of a label free Lab-on-Chip platform that can genotype breast cancer variants rapidly to assist tumour progression surveillance and individuality of BC in the clinic as an affordable alternative to NGS and ddPCR.

## Materials and Methods

### Samples and DNA extraction methods

Two plasmids, purchased from ThermoFisher Scientific (United States), were utilised as the synthetic DNA material for validation of the developed primer sets. Both plasmids consisted of PUC 18 cloning vectors containing a fragment of interest of 531 bp harbouring the SNV p.H1047R. The plasmid harbouring the WT allele p.H1047R is named as WT template, and the plasmid harbouring the MT allele p.H1047R is named as MT template. On the other hand, DNA was extracted from patient FFPE tissue cores using the Qiagen GeneRead Kit according to manufacturer’s instructions and quantified using Qubit fluorometer. DNA was extracted from a healthy control tissue core as a WT control (H1047R negative) and from a tumour patient core (H1047R heterozygous sample). Patient samples used in this study were from our recent paper *Coombes et al*.^31^ with the trial protocol approved by the Riverside Research Ethics Committee REC:13/LO/115; IRAS:126462. Methods were carried out in accordance with the relevant guidelines and regulations. Informed consent was obtained from all participants and/or their legal guardian/s.

### Sanger sequencing

The T47D cell line was genotyped by PCR amplification using 30 ng of DNA, followed by Sanger Sequencing using an ABI 3730 automatic genetic analyser (Applied Biosystems, Foster City, CA). Primers sequences included: forward, 5′- AGAACTACAATCTTTTGATGACA -3′, and reverse, 5′-TGGAATCCAGAGTGAGCT-3′. The sequence reads were analysed using the sequencing analysis tools in Geneious^35^.

### Droplet Digital PCR

Validation of the *PIK3CA* p.H1047R mutation was performed using a Bio-Rad QX200 droplet digital PCR system as described previously^36^. Primer sequences included: forward, 5′-AGAGGCTTTGGAGTATTTCATG-3′; reverse, 5′-TGCATGCTGTTTAATTGTGTG-3′; probe sequences were wild-type VIC-MGB 5′-CCACCATGATGTGCA-3′; and mutant FAM-MGB 5′-CCACCATGACGTGCA-3′. The optimum melting temperature for this assay was 62 °C. The assay was designed using OligoArchitect. For each assay 10 ng of FFPE tissue DNA was run alongside 10 ng of hgDNA (negative control), 10 ng TD47 cell line DNA (heterozygous control) and NTC (no template control).

### SNV-specific primer design for detecting p.H1047R

Consensus reference genomic sequence from human gene *PIK3CA* (Gene ID: 5290) was retrieved from National Centre for Biotechnology Information (NCBI)^37^ and analysed for specific target regions among biological sequences using BLAST^38^. Following the guidelines provided by *Malpartida-Cardenas et al*.^15^ for the design of USS-sbLAMP primers, two primer sets were designed targeting the SNV p.H1047R within the gene *PIK3CA*. Each primer set consisted of 8 primers, being 6 of them responsible for SNV-based loop mediated isothermal amplification (sbLAMP) including sbFIP and sbBIP which are in charge of the allele-specificity, and 2 unmodified self-stabilising (USS) primers, FB and BB, responsible for delaying or preventing unspecific sbLAMP amplification. The sbLAMP primers were designed using Primer Explorer V5 (Eiken Chemical Co. Ltd., Tokyo, Japan; http://primerexplorer.jp/lampv5e/index.html) and LAMP guidelines^14^ to obtain F3, B3, F2 and B2 priming regions, and optimized manually to locate both loop primers LF and LB, and the SNV at the 5′ end of F1c and B1c within sbFIP and sbBIP. USS primers were designed following the method described by *Malpartida-Cardenas et al*.^15^ based on the most optimal sbFIP and sbBIP tested experimentally. The primer set specifically detecting the WT allele p.1047 H, named as USS_MT_-sbLAMP_WT_ (WT reaction), consisted of F1c_19_-B1c_14_ + FB2_21_/BB2_24_ at 3 µM; the primer set specifically detecting the MT allele, named as USS_WT_-sbLAMP_MT_ (MT reaction), consisted of F1c_17_-B1c_14_ + FB2_19_/BB1_23_ at 3 µM.

### Reaction conditions

Each LAMP reaction mixture contained the following: 1.5 μL of 10x isothermal buffer, 0.9 μL of MgSO4 (100 mM stock), 2.1 μL of dNTPs (10 mM stock), 0.375 μL of BSA (20 mg/mL stock), 2.4 μL of Betaine (5 M stock), 0.375 μL of SYTO 9 Green (20 μM stock), 0.6 μL of Bst 2.0 DNA polymerase (8,000 U/mL stock), 3 μL of different concentrations of synthetic plasmid DNA, 1.5 μL of $10x$ USS-sbLAMP primer mixture (20 μM sbBIP/sbFIP, 10 μM LF/LB, 2.5 μM B3/F3 and 30 μM of FB/BB) and enough nuclease-free water (ThermoFisher Scientific) to bring the volume to 15 µL. All reagents were purchased from New England BioLabs (United Kingdom). Reactions were performed at 63 °C for 35 min. Two reactions, WT reaction and MT reaction, were performed independently. Experiments were performed twice with each condition in triplicate (5 μL each reaction) utilising LightCycler 480 Multiwell Plates 96 (Roche Diagnostics) and a LightCycler 96 Real-Time PCR System (Roche Diagnostics. In the case of pH-LAMP, the buffering conditions were modified such that pH changes could be measured. Each reaction contained the following: 3.0 μL of 10x isothermal customized buffer (pH 8.5–9), 1.8 μL of MgSO4 (100 mM stock), 4.2 μL of dNTPs (10 mM stock), 1.8 μL of BSA (20 mg/mL stock), 4.8 μL of Betaine (5 M stock), 1.88 μL of Bst 2.0 DNA polymerase (8,000 U/mL stock), 3 μL of different concentrations of synthetic DNA or gDNA, 0.75 μL of NaOH (0.2 M stock), 3 μL of 10x LAMP primer mixture, 0.75 μL of SYTO 9 Green (20 μM stock) only for qPCR experiments, and enough nuclease-free water (ThermoFisher Scientific) to bring the volume to 30 μL. Reactions tested on the microchip contained 13 μL each. sbLAMP and USS primers were purchased from Integrated DNA Technologies (The Netherlands) and resuspended in TE buffer to 100 µM and 400 µM stock solutions, respectively. The solutions were stored at 4 °C.

### Analytical sensitivity and specificity of USS-sbLAMP primer sets for detection of SNV p.H1047R

Analytical sensitivity was tested using ten-fold serial dilutions (1 × 10^7^, 1 × 10^6^, 1 × 10^5^, 1 × 10^4^ and 1 × 10^3^ copies/reaction which are equivalent to 31.356, 3.136, 0.314, 0.031 and 0.003 pg/reaction) of WT and MT templates, independently. Standard curves were obtained by plotting the TTP values with errors at one standard deviation against their corresponding DNA concentrations. Sensitivity including background DNA was also assessed by including in the reaction mixture Salmon testes sonicated DNA (Sigma-Aldrich) at 13 ng/reaction. Sensitivity of mixed populations was evaluated by spiking WT and MT templates at different ratios (100/0, 80/20, 50/50, 20/80 and 0/100, in percentage) to a final concentration of 1 × 10^7^ copies/reaction.

### SNV-specific LAMP on a microchip-based Lab-on-Chip platform

Electrochemical sensing for the detection of the pH changes induced during pH-LAMP is facilitated using ISFETs fabricated in unmodified complementary metal-oxide-semiconductor (CMOS) technology. ISFETs detect changes in the concentration of hydrogen ions at the passivation layer of the microchip through the modulation of an induced voltage across that layer^22,39^. This way, owing to the compatibility of fabrication in standard CMOS technology allows for the mass manufacturing, low cost, and miniaturisation of sensors while ensuring non-optical and fully-electronic chemical (pH) detection.

The Lab-on-Chip platform used here comprises an array of 64 × 64 ISFET sensors fabricated in the AMS 0.35 ${\boldsymbol{\mu }}{\boldsymbol{m}}$ CMOS process using silicon nitride $({\boldsymbol{S}}{{\boldsymbol{i}}}_{3}{{\boldsymbol{N}}}_{4})$ as the passivation (sensing) layer. The total sensing area spans 0.56 ${\boldsymbol{m}}{{\boldsymbol{m}}}^{2}$ with an input-output pH sensitivity of 9.23 mV/pH. A detailed description of the circuit characteristics and additional specification is provided in *N. Miscourides et al*.^16^. Furthermore, a custom microfluidic manifold was added on top of the microchip to host the reaction mix during LAMP, laser cut from plastic acrylic sheets. Thermal control for DNA amplification is facilitated using an external Verity Thermal Cycler instrument.

### Statistical analysis

Data is presented in minutes as TTP ± error at one standard deviation. Displayed p-values were calculated using a two-sided distribution Student’s heteroscedastic t-test; statistical significance was considered as following: *p-value < 0.05, **p-value < 0.01, ***p-value < 0.001, ****p-value < 0.0001.

## Data Availability

All data generated or analysed during this study are included in this published article. Data generated and analysed throughout the study can be made available from the corresponding author on reasonable request.
